# (2,2′-Bipyridine-κ^2^ *N*,*N*′)iodido(piperidine-1-carbodithio­ato-κ^2^ *S*,*S*′)copper(II)

**DOI:** 10.1107/S1600536808039901

**Published:** 2008-12-03

**Authors:** Le-Qing Fan

**Affiliations:** a Institute of Materials Physical Chemistry and The Key Laboratory for Functional Materials of Fujian Higher Education, Huaqiao University, Quanzhou, Fujian 362021, People’s Republic of China

## Abstract

In the title compound, [Cu(C_6_H_10_NS_2_)I(C_10_H_8_N_2_)], the Cu^II^ ion is coordinated by one iodide ion, two N atoms of the bipyridine ligand and two S atoms from the piperidine­carbodithio­ate ligand in a distorted square-pyramidal environment. π–π stacking inter­actions, with centroid–centroid distances of 3.643 (4) Å, between pyridyl rings of the bipyridyl ligands of neighbouring mol­ecules lead to chains propagating parallel to the *a* axis.

## Related literature

For background to transition metal complexes, see: Engelhardt *et al.* (1988 ▶); Fernández *et al.* (2000 ▶); Koh *et al.* (2003 ▶); Noro *et al.* (2000 ▶); Yaghi *et al.* (1998 ▶).
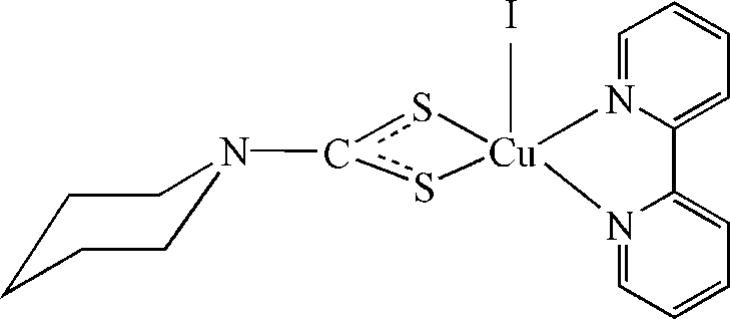


## Experimental

### 

#### Crystal data

[Cu(C_6_H_10_NS_2_)I(C_10_H_8_N_2_)]
*M* *_r_* = 506.89
Monoclinic, 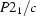

*a* = 6.532 (3) Å
*b* = 16.859 (7) Å
*c* = 17.578 (7) Å
β = 108.047 (14)°
*V* = 1840.5 (14) Å^3^
*Z* = 4
Mo *K*α radiation
μ = 3.09 mm^−1^
*T* = 293 (2) K
0.45 × 0.08 × 0.05 mm

#### Data collection

Rigaku Mercury CCD diffractometer
Absorption correction: multi-scan (*CrystalClear*; Rigaku, 2000 ▶) *T*_min_ = 0.751, *T*_max_ = 1.000 (expected range = 0.643–0.857)
13746 measured reflections
3946 independent reflections
3389 reflections with *I* > 2σ(*I*)
*R*_int_ = 0.036

#### Refinement

*R*[*F* ^2^ > 2σ(*F* ^2^)] = 0.047
*wR*(*F* ^2^) = 0.108
*S* = 1.08
3946 reflections
208 parameters
H-atom parameters constrained
Δρ_max_ = 0.52 e Å^−3^
Δρ_min_ = −0.63 e Å^−3^

### 

Data collection: *CrystalClear* (Rigaku, 2000 ▶); cell refinement: *CrystalClear*; data reduction: *CrystalClear*; program(s) used to solve structure: *SHELXS97* (Sheldrick, 2008 ▶); program(s) used to refine structure: *SHELXL97* (Sheldrick, 2008 ▶); molecular graphics: *SHELXTL* (Sheldrick, 2008 ▶); software used to prepare material for publication: *SHELXTL*.

## Supplementary Material

Crystal structure: contains datablocks global, I. DOI: 10.1107/S1600536808039901/ez2153sup1.cif

Structure factors: contains datablocks I. DOI: 10.1107/S1600536808039901/ez2153Isup2.hkl

Additional supplementary materials:  crystallographic information; 3D view; checkCIF report

## Figures and Tables

**Fig. 1.**
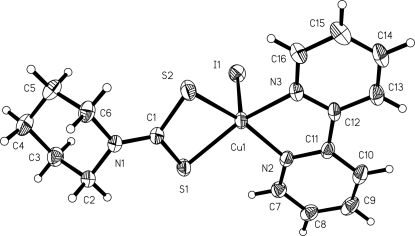
The molecular structure of (I), showing the atom-labelling scheme, with 30% probability displacement ellipsoids.

## supplementary crystallographic information

### Comment

Research into transition metal complexes has been rapidly expanding because of their fascinating structural diversity, as well as their potential applications as functional materials and enzymes (Noro *et al.*, 2000; Yaghi *et al.*, 1998). Dialkyldithiocarbamate anions, which are typical sulfur ligands, acting as monodentate, bidentate or bridging ligands, are often chosen for the preparation of complexes with a considerable structural variety (Engelhardt *et al.*, 1988; Fernández *et al.*, 2000; Koh *et al.*, 2003). I report here the crystal structure of the title copper(II) complex, (I), containing a piperidyldithiocarbamate ligand.

The crystal structure of (I) is built of discrete molecules of the Cu^II^ complex (Fig. 1). The Cu^II^ ion is five-coordinated in a distorted square-pyramidal environment by one I atom in the apical position, two N atoms from the bipyridine ligand and two S atoms from the piperidyldithiocarbamate ligand in the basal plane (Table 1).

There is a π-π stacking interaction between the pyridyl rings R1 [N(2)/C(7)–C(11)] and R2 [N3/C(12)–C(16)] with a centroid-to-centroid distance of 3.643 (4) Å. These face-to-face interactions result in the complexes assembling into chains.

### Experimental

A mixture of Cu(Ac)_2_.H_2_O (0.08 g, 0.4 mmol), NaS_2_CNC_5_H_10_.2H_2_O (0.09 g, 0.4 mmol), 2,2'-bipyridine (0.06 g 0.4 mmol) and NaI.2H_2_O (0.07 g, 0.4 mmol) was stirred in DMF (15 ml). 2-PrOH was diffused into the resulting solution, yielding single crystals of (I).

### Refinement

H atoms were positioned geometrically and refined as riding atoms, with C—H = 0.93 (aromatic) or 0.97 Å (piperidyl); *U*_iso_(H) = 1.2*U*_eq_(C).

### Crystal data

**Table:**

[Cu(C_6_H_10_NS_2_)I(C_10_H_8_N_2_)]	*F*(000) = 996
*M**_r_* = 506.89	*D*_x_ = 1.829 Mg m^−^^3^
Monoclinic, *P*2_1_/*c*	Mo *K*α radiation, λ = 0.71073 Å
Hall symbol: -P 2ybc	Cell parameters from 3988 reflections
*a* = 6.532 (3) Å	θ = 3.3–27.5°
*b* = 16.859 (7) Å	µ = 3.09 mm^−^^1^
*c* = 17.578 (7) Å	*T* = 293 K
β = 108.047 (14)°	Prism, black
*V* = 1840.5 (14) Å^3^	0.45 × 0.08 × 0.05 mm
*Z* = 4	

### Data collection

**Table:**

Rigaku Mercury CCD diffractometer	3946 independent reflections
Radiation source: Sealed Tube	3389 reflections with *I* > 2σ(*I*)
Graphite Monochromator	*R*_int_ = 0.036
ω scans	θ_max_ = 27.5°, θ_min_ = 2.4°
Absorption correction: multi-scan (*CrystalClear*; Rigaku,2000)	*h* = −8→6
*T*_min_ = 0.751, *T*_max_ = 1.000	*k* = −21→21
13746 measured reflections	*l* = −22→22

### Refinement

**Table:**

Refinement on *F*^2^	Primary atom site location: structure-invariant direct methods
Least-squares matrix: full	Secondary atom site location: difference Fourier map
*R*[*F*^2^ > 2σ(*F*^2^)] = 0.047	Hydrogen site location: inferred from neighbouring sites
*wR*(*F*^2^) = 0.108	H-atom parameters constrained
*S* = 1.09	*w* = 1/[σ^2^(*F*_o_^2^) + (0.043*P*)^2^ + 2.8277*P*] where *P* = (*F*_o_^2^ + 2*F*_c_^2^)/3
3946 reflections	(Δ/σ)_max_ = 0.001
208 parameters	Δρ_max_ = 0.52 e Å^−^^3^
0 restraints	Δρ_min_ = −0.63 e Å^−^^3^

### Special details

**Table:**

Geometry. All e.s.d.'s (except the e.s.d. in the dihedral angle between two l.s. planes) are estimated using the full covariance matrix. The cell e.s.d.'s are taken into account individually in the estimation of e.s.d.'s in distances, angles and torsion angles; correlations between e.s.d.'s in cell parameters are only used when they are defined by crystal symmetry. An approximate (isotropic) treatment of cell e.s.d.'s is used for estimating e.s.d.'s involving l.s. planes.
Refinement. Refinement of *F*^2^ against ALL reflections. The weighted *R*-factor *wR* and goodness of fit *S* are based on *F*^2^, conventional *R*-factors *R* are based on *F*, with *F* set to zero for negative *F*^2^. The threshold expression of *F*^2^ > σ(*F*^2^) is used only for calculating *R*-factors(gt) *etc*. and is not relevant to the choice of reflections for refinement. *R*-factors based on *F*^2^ are statistically about twice as large as those based on *F*, and *R*- factors based on ALL data will be even larger.

### Fractional atomic coordinates and isotropic or equivalent isotropic displacement parameters (Å^2^)

**Table:**

	*x*	*y*	*z*	*U*_iso_*/*U*_eq_	
Cu1	0.43714 (10)	0.66212 (3)	0.42086 (3)	0.04099 (17)	
I1	0.19321 (6)	0.80141 (2)	0.36188 (2)	0.05420 (14)	
S1	0.6396 (2)	0.64039 (8)	0.33518 (8)	0.0502 (3)	
S2	0.2619 (3)	0.55763 (9)	0.34096 (8)	0.0586 (4)	
N1	0.4731 (7)	0.5184 (2)	0.2378 (2)	0.0423 (9)	
N2	0.6611 (7)	0.7232 (2)	0.5076 (2)	0.0401 (9)	
N3	0.3271 (7)	0.6421 (2)	0.5154 (2)	0.0420 (9)	
C1	0.4607 (8)	0.5651 (3)	0.2965 (3)	0.0386 (10)	
C2	0.6306 (8)	0.5319 (3)	0.1943 (3)	0.0496 (12)	
H2A	0.7044	0.4828	0.1907	0.059*	
H2B	0.7370	0.5706	0.2225	0.059*	
C3	0.5104 (9)	0.5620 (3)	0.1113 (3)	0.0556 (14)	
H3A	0.6106	0.5693	0.0811	0.067*	
H3B	0.4459	0.6130	0.1154	0.067*	
C4	0.3347 (10)	0.5035 (4)	0.0674 (3)	0.0620 (15)	
H4A	0.2530	0.5255	0.0160	0.074*	
H4B	0.4004	0.4544	0.0578	0.074*	
C5	0.1852 (9)	0.4865 (3)	0.1159 (3)	0.0533 (13)	
H5A	0.1057	0.5342	0.1194	0.064*	
H5B	0.0822	0.4461	0.0891	0.064*	
C6	0.3075 (9)	0.4585 (3)	0.1993 (3)	0.0499 (13)	
H6A	0.2094	0.4515	0.2304	0.060*	
H6B	0.3757	0.4079	0.1966	0.060*	
C7	0.8254 (8)	0.7641 (3)	0.4972 (3)	0.0464 (12)	
H7A	0.8406	0.7663	0.4464	0.056*	
C8	0.9733 (9)	0.8031 (3)	0.5591 (3)	0.0537 (13)	
H8A	1.0845	0.8323	0.5503	0.064*	
C9	0.9520 (10)	0.7979 (3)	0.6347 (4)	0.0615 (16)	
H9A	1.0515	0.8226	0.6777	0.074*	
C10	0.7829 (9)	0.7558 (3)	0.6462 (3)	0.0553 (14)	
H10A	0.7675	0.7519	0.6969	0.066*	
C11	0.6367 (8)	0.7197 (3)	0.5814 (3)	0.0430 (11)	
C12	0.4453 (9)	0.6746 (3)	0.5851 (3)	0.0417 (11)	
C13	0.3853 (10)	0.6676 (3)	0.6542 (3)	0.0528 (13)	
H13A	0.4662	0.6917	0.7016	0.063*	
C14	0.2037 (10)	0.6243 (3)	0.6510 (3)	0.0587 (15)	
H14A	0.1636	0.6173	0.6970	0.070*	
C15	0.0828 (10)	0.5918 (3)	0.5800 (4)	0.0614 (16)	
H15A	−0.0417	0.5634	0.5769	0.074*	
C16	0.1483 (9)	0.6016 (3)	0.5129 (3)	0.0515 (13)	
H16A	0.0661	0.5794	0.4646	0.062*	

### Atomic displacement parameters (Å^2^)

**Table:**

	*U*^11^	*U*^22^	*U*^33^	*U*^12^	*U*^13^	*U*^23^
Cu1	0.0480 (4)	0.0472 (3)	0.0298 (3)	−0.0078 (3)	0.0150 (3)	−0.0058 (2)
I1	0.0605 (3)	0.0538 (2)	0.0489 (2)	0.00754 (16)	0.01783 (18)	0.01277 (15)
S1	0.0491 (8)	0.0629 (8)	0.0430 (6)	−0.0169 (6)	0.0207 (6)	−0.0195 (6)
S2	0.0670 (10)	0.0688 (8)	0.0495 (7)	−0.0287 (7)	0.0322 (7)	−0.0201 (6)
N1	0.043 (3)	0.048 (2)	0.0346 (19)	−0.0025 (18)	0.0103 (18)	−0.0079 (16)
N2	0.043 (3)	0.0428 (19)	0.0308 (18)	0.0023 (17)	0.0068 (17)	−0.0013 (15)
N3	0.057 (3)	0.0387 (19)	0.0343 (19)	0.0032 (18)	0.0195 (18)	0.0019 (15)
C1	0.044 (3)	0.040 (2)	0.031 (2)	−0.002 (2)	0.011 (2)	−0.0012 (17)
C2	0.043 (3)	0.062 (3)	0.042 (3)	−0.001 (2)	0.011 (2)	−0.017 (2)
C3	0.060 (4)	0.066 (3)	0.042 (3)	−0.010 (3)	0.019 (3)	−0.003 (2)
C4	0.069 (4)	0.070 (4)	0.038 (3)	−0.011 (3)	0.003 (3)	−0.004 (3)
C5	0.056 (4)	0.045 (3)	0.047 (3)	−0.010 (2)	−0.001 (3)	−0.001 (2)
C6	0.065 (4)	0.040 (2)	0.042 (3)	−0.012 (2)	0.013 (2)	−0.007 (2)
C7	0.047 (3)	0.048 (3)	0.044 (3)	−0.005 (2)	0.013 (2)	−0.002 (2)
C8	0.048 (3)	0.048 (3)	0.058 (3)	−0.002 (2)	0.005 (3)	−0.008 (2)
C9	0.060 (4)	0.053 (3)	0.054 (3)	0.001 (3)	−0.008 (3)	−0.019 (3)
C10	0.067 (4)	0.052 (3)	0.037 (3)	0.012 (3)	0.003 (3)	−0.007 (2)
C11	0.050 (3)	0.044 (2)	0.030 (2)	0.014 (2)	0.006 (2)	−0.0001 (18)
C12	0.058 (3)	0.042 (2)	0.027 (2)	0.012 (2)	0.016 (2)	0.0019 (17)
C13	0.071 (4)	0.054 (3)	0.037 (3)	0.017 (3)	0.022 (3)	0.010 (2)
C14	0.085 (5)	0.058 (3)	0.046 (3)	0.021 (3)	0.038 (3)	0.013 (2)
C15	0.082 (4)	0.046 (3)	0.073 (4)	0.007 (3)	0.048 (3)	0.006 (3)
C16	0.067 (4)	0.045 (3)	0.052 (3)	−0.004 (2)	0.033 (3)	−0.004 (2)

### Geometric parameters (Å, °)

**Table:**

Cu1—N3	2.033 (4)	C5—C6	1.510 (7)
Cu1—N2	2.035 (4)	C5—H5A	0.9700
Cu1—S1	2.3205 (15)	C5—H5B	0.9700
Cu1—S2	2.3218 (15)	C6—H6A	0.9700
Cu1—I1	2.8470 (11)	C6—H6B	0.9700
S1—C1	1.717 (5)	C7—C8	1.379 (7)
S2—C1	1.716 (5)	C7—H7A	0.9300
N1—C1	1.320 (6)	C8—C9	1.380 (8)
N1—C2	1.476 (6)	C8—H8A	0.9300
N1—C6	1.482 (6)	C9—C10	1.379 (9)
N2—C7	1.335 (6)	C9—H9A	0.9300
N2—C11	1.357 (6)	C10—C11	1.380 (7)
N3—C16	1.342 (6)	C10—H10A	0.9300
N3—C12	1.345 (6)	C11—C12	1.481 (7)
C2—C3	1.514 (7)	C12—C13	1.392 (6)
C2—H2A	0.9700	C13—C14	1.380 (8)
C2—H2B	0.9700	C13—H13A	0.9300
C3—C4	1.529 (7)	C14—C15	1.369 (8)
C3—H3A	0.9700	C14—H14A	0.9300
C3—H3B	0.9700	C15—C16	1.384 (7)
C4—C5	1.511 (8)	C15—H15A	0.9300
C4—H4A	0.9700	C16—H16A	0.9300
C4—H4B	0.9700		
			
N3—Cu1—N2	79.95 (16)	C6—C5—C4	111.5 (5)
N3—Cu1—S1	157.11 (12)	C6—C5—H5A	109.3
N2—Cu1—S1	98.30 (12)	C4—C5—H5A	109.3
N3—Cu1—S2	97.80 (12)	C6—C5—H5B	109.3
N2—Cu1—S2	160.43 (12)	C4—C5—H5B	109.3
S1—Cu1—S2	76.17 (5)	H5A—C5—H5B	108.0
N3—Cu1—I1	97.76 (11)	N1—C6—C5	108.8 (4)
N2—Cu1—I1	92.69 (11)	N1—C6—H6A	109.9
S1—Cu1—I1	105.13 (5)	C5—C6—H6A	109.9
S2—Cu1—I1	106.86 (6)	N1—C6—H6B	109.9
C1—S1—Cu1	85.37 (16)	C5—C6—H6B	109.9
C1—S2—Cu1	85.37 (16)	H6A—C6—H6B	108.3
C1—N1—C2	122.3 (4)	N2—C7—C8	122.3 (5)
C1—N1—C6	123.4 (4)	N2—C7—H7A	118.8
C2—N1—C6	113.3 (4)	C8—C7—H7A	118.8
C7—N2—C11	119.4 (4)	C7—C8—C9	118.3 (6)
C7—N2—Cu1	125.5 (3)	C7—C8—H8A	120.8
C11—N2—Cu1	115.1 (3)	C9—C8—H8A	120.8
C16—N3—C12	119.0 (4)	C10—C9—C8	119.9 (5)
C16—N3—Cu1	125.6 (3)	C10—C9—H9A	120.1
C12—N3—Cu1	115.4 (3)	C8—C9—H9A	120.1
N1—C1—S2	123.5 (4)	C9—C10—C11	119.2 (5)
N1—C1—S1	123.5 (4)	C9—C10—H10A	120.4
S2—C1—S1	113.1 (2)	C11—C10—H10A	120.4
N1—C2—C3	108.3 (4)	N2—C11—C10	120.9 (5)
N1—C2—H2A	110.0	N2—C11—C12	114.5 (4)
C3—C2—H2A	110.0	C10—C11—C12	124.7 (5)
N1—C2—H2B	110.0	N3—C12—C13	121.5 (5)
C3—C2—H2B	110.0	N3—C12—C11	115.1 (4)
H2A—C2—H2B	108.4	C13—C12—C11	123.4 (5)
C2—C3—C4	110.8 (4)	C14—C13—C12	118.7 (5)
C2—C3—H3A	109.5	C14—C13—H13A	120.6
C4—C3—H3A	109.5	C12—C13—H13A	120.6
C2—C3—H3B	109.5	C15—C14—C13	119.6 (5)
C4—C3—H3B	109.5	C15—C14—H14A	120.2
H3A—C3—H3B	108.1	C13—C14—H14A	120.2
C5—C4—C3	110.6 (4)	C14—C15—C16	119.1 (6)
C5—C4—H4A	109.5	C14—C15—H15A	120.4
C3—C4—H4A	109.5	C16—C15—H15A	120.4
C5—C4—H4B	109.5	N3—C16—C15	121.9 (5)
C3—C4—H4B	109.5	N3—C16—H16A	119.1
H4A—C4—H4B	108.1	C15—C16—H16A	119.1
